# Genetic testing for hearing loss in the United States should include deletion/duplication analysis for the deafness/infertility locus at 15q15.3

**DOI:** 10.1186/1755-8166-6-19

**Published:** 2013-05-06

**Authors:** Nicole Hoppman, Umut Aypar, Pamela Brodersen, Neil Brown, Justin Wilson, Dusica Babovic-Vuksanovic

**Affiliations:** 1 Department of Laboratory Medicine and Pathology, Mayo Clinic, 971 Hilton, 200 1st St SW, Rochester, MN, 55905, USA; 2 Department of Otorhinolaryngology, Mayo Clinic Health System, La Crosse, WI, USA; 3 Department of Medical Genetics, Mayo Clinic, Rochester, MN, USA

**Keywords:** Deafness-Infertility Syndrome, CATSPER2, STRC, Array CGH

## Abstract

**Background:**

Hearing loss is the most common birth defect and the most prevalent sensorineural disorder in developed countries. More than 50% of prelingual deafness is genetic, most often autosomal recessive and nonsyndromic, of which 50% can be attributed to the disorder DFNB1, caused by mutations in *GJB2* and *GJB6.* Sensorineural hearing loss and male infertility (Deafness-Infertility Syndrome; DIS) is a contiguous gene deletion syndrome resulting from homozygous deletion of the *CATSPER2* and *STRC* genes on chromosome 15q15.3. Females with DIS have only hearing loss and are fertile. Until recently this syndrome has only been described in three consanguineous families and 2 nonconsanguineous families.

**Results:**

We recently indentified a patient with hearing loss and macrocephaly who was found to be homozygous for this deletion. Her nonconsanguineous parents are both carriers. We examined our database of patients tested by array CGH and determined that just over 1% of our patients are heterozygous for this deletion. If this number is representative of the general population, this implies a 1% carrier frequency and prevalence of DIS of 1 in 40,000 individuals.

**Conclusion:**

We propose that DIS is a greatly under-diagnosed cause of deafness and should be considered in children with hearing loss. Likewise, current molecular genetic testing panels for hearing loss in the United States should be expanded to include deletion/duplication analysis of this region.

## Background

Hearing loss is the most common birth defect and the most prevalent sensorineural disorder in developed countries. One out of every 500 newborns has permanent bilateral sensorineural hearing loss ≥40 dB; by adolescence, the prevalence increases to 3.5 per 1000 [1]. More than 50% of prelingual deafness is genetic, most often autosomal recessive and nonsyndromic, while only a small percentage of prelingual deafness is syndromic or autosomal dominant nonsyndromic. Approximately 50% of autosomal recessive nonsyndromic hearing loss can be attributed to the disorder DFNB1, caused by mutations in *GJB2* (which encodes the protein connexin 26) and *GJB6* (which encodes the protein connexin 30) [2]. The carrier rate in the general population for a recessive deafness-causing *GJB2* mutation is approximately one in 33 [2].

Sensorineural hearing loss and male infertility (Deafness-Infertility Syndrome; DIS) is a contiguous gene deletion syndrome resulting from homozygous deletion of the *CATSPER2* (responsible for male infertility) and *STRC* (responsible for hearing loss) genes on chromosome 15q15.3. Females with DIS have only hearing loss and are fertile [3]. Until recently, this syndrome had only been described in three consanguineous families [4] and two individuals with unrelated parents [5-7]. However, a patient with nonconsanguineous parents who had hearing loss and macrocephaly presented to the Medical Genetics department at Mayo Clinic recently and was found to be homozygous for this deletion, resulting in DIS. Both were found to be carriers. We then decided to retrospectively examine our database of patients tested by array CGH in order to determine the frequency of this deletion in our patient population.

## Results

The proband is a 13-month-old girl who presented to the Medical Genetics Department for evaluation regarding congenital hearing loss. Her past medical history was unremarkable. She was the second child born to a 35-year-old mother. Except for morning nausea the pregnancy was not complicated. The mother did not take any medications during gestation. Delivery was at term (40 weeks), birth weight was 3856 grams (75th centile), and no perinatal complications were reported. Her psychomotor development has been normal. The patient was crawling at age 7 months, standing up at age 8 months, and walked unassisted at age 10 months. Her language at age 13 months was appropriately limited to babbling and a few words.

**Figure 1:**
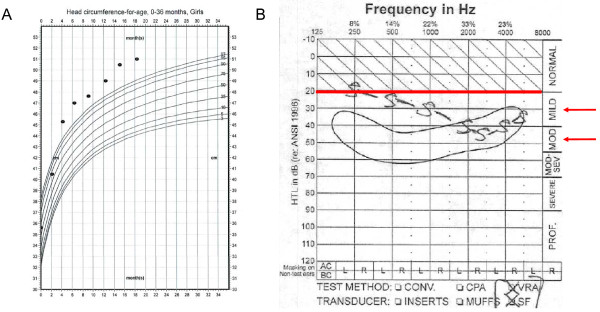
Phenotypic features of the proband, demonstrating macrocephaly from age 4 months on (A) and mild-moderate hearing loss in the 500–6000 Hz range (B).

At age 13 months, her physical examination was normal except for the presence of macrocephaly. Her height was 75 cm (70th centile), and her weight was 12.5 kg (94th centile). Her head circumference was 49.7 cm (>99th centile). In addition to macrocephaly, anterior fontanel was still open (2–2 cm) and there was mild brachycephaly, but the face was non-dysmorphic. A review of her growth chart revealed that she had normal head circumference at birth (35 cm, 56th centile), but her head size exceeded the 99th centile at age 4 months. Since that time, her head circumference has consistently tracked above the 99th centile (Figure 1A). The patient does not have a history of seizures or any other neurological deficits, and there is also no family history of macrocephaly.

With regard to the patient’s hearing loss, she failed the initial newborn hearing screen in both ears. Follow-up diagnostic testing via high- frequency tympanometry, otoacoustic emissions (both transient & distortion product), and auditory brainstem response (ABR) suggested normal middle ear function, good 8th nerve synchrony, but a mild hearing loss at least for the 2000–4000 Hz range in both ears. The hearing loss was most likely sensorineural in nature given the normal middle ear function (evidenced by tympanometry) with absent otoacoustic emissions. Behavioral audiological testing via visual reinforcement audiometry in the sound-field was first completed at 8 months of age and has continued at 6 months intervals. Behavioral testing verified the hearing loss predicted by ABR and has suggested some progression to date. The patient’s most recent test results suggested a mild sloping to moderate hearing loss (most likely sensorineural in type) involving the 500–6000 Hz range for at least the better ear but involving both ears (Figure 1B). Family history is negative for early onset of hearing loss, and her parents are not consanguineous.

**Figure 2:**
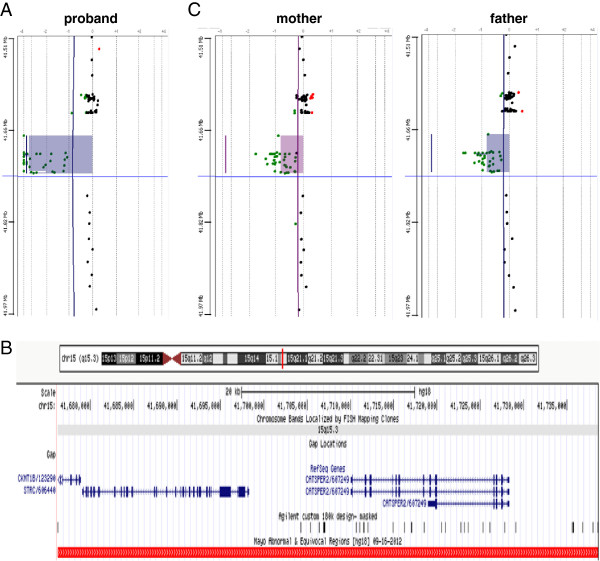
**Array CGH results demonstrating a homozygous deletion on 15q15.3 in the proband (A) that includes the full *STRC *and *CATSPER2 *genes as well as a portion of *CKMT1B *as shown by the red rectangle (B).** Both parents were found to be heterozygous carriers of this deletion (**C**).

In the context of mild macrocephaly, delayed closure of fontanelles and underlying hearing loss, we considered a number of diagnostic possibilities including peroxisomal disorders, mannosidosis, organic acidemias and chromosomal alterations. The initial diagnostic workup included urinalysis and plasma peroxisomal panel, which were normal except for marginal elevation of phytanic acid. Sequencing of *GJB2* and deletion/duplication testing of *GJB6* were also negative. However, array comparative genomic hybridization (array CGH) detected an approximately 62 kilobase homozygous deletion at 15q15.3 (human genome build hg18 coordinates 41,676,219-41,738,593, Figure 2A). The deleted interval contains the entire *STRC* and *CATSPER2* genes, as well as a portion of *CKMT1B* (Figure 2B). Parental array CGH testing demonstrated that both parents are carriers of a heterozygous deletion at 15q15.3 (Figure 2C).

Because of the paucity of previously reported patients in the literature, along with the fact that three were reportedly from consanguineous unions, we decided to examine the frequency of this deletion in our database of patient samples. In 2011, our laboratory tested 5,152 patient samples (peripheral blood specimens) by array CGH. Of those, 57 patients were found to be heterozygous for similar deletions including the *STRC* and *CATSPER2* genes, two of whom were siblings, indicating that 1.09% of patients are carriers. If this figure is representative of the general population, this would indicate that approximately 1 in 40,000 individuals is born with a homozygous deletion of this region, resulting in deafness and, in males, infertility.

## Discussion

We present here the third reported case of a patient with DIS whose parents were not consanguineous (Table 1). In this particular situation, because the patient presented with macrocephaly in addition to hearing loss, an array CGH was ordered, which is how the homozygous deletion of 15q15.3 was detected. However, in the absence of any other phenotypic findings, array CGH is typically not performed in children with isolated hearing loss. This means that females with 15q15.3 homozygous deletion, who have only deafness, would not have an array CGH ordered. Males with DIS have infertility in addition to hearing loss; however these individuals are most likely evaluated during childhood (before the infertility phenotype would be apparent) and therefore do not routinely have array CGH performed for diagnostic purposes. Currently, array CGH is the only clinical test available that can detect deletions resulting in DIS in the United States. Given the presence of a heterozygous deletion of this region in just over 1% of our patient population (hence polymorphic), we hypothesize that DIS is an under-diagnosed cause of hearing loss.

**Table 1:** Summary of DIS patients with homozygous 15q15.3 microdeletion

**Publication**	**Patient gender**	**Patient age**	**Phenotype**	**Consanguinity?**
**Zhang et al. (family D_SM)**	Male (n = 4)	23 and 35 years, not provided for rest	All have sensorineural HL and no syndromic features; one male had sperm motility assessed: asthenoteratozoospermia	Yes
**Zhang et al. (family L705)**	Female (n = 2), male (n = 1)	Females 35 and 20 years, male not provided	All have sensorineural HL and no syndromic features; sperm motility assessment not performed	Yes
**Zhang et al. (family L1014)**	Female (n = 1), male (n = 2)	Males: 26 and 21 years; female 17 years	All have sensorineural HL and no syndromic features; both males have asthenoteratozoospermia	Yes
**Avidan et al.**	Male	56 years	Moderate sensurineural HL, infertility (asthenoteratozoospermia)	No; two brothers with similar phenotype
**Knijnenburg et al.**	Male	10 years	Moderate, bilateral sensorineural HL, MR, short stature, dysmorphic features, normal HC, sperm motility assessment not performed	No
**This publication**	Female	13 months	Mild/moderate bilateral sensorineural HL, macrocephaly	No

Abbreviations: *HL* Hearing Loss, *MR* Mental Retardation, *HC* Head Circumference.

One important limitation to point out is that our study cohort is comprised of individuals referred to clinics for a variety of reasons, including but not limited to developmental delay, dysmorphism, congenital anomalies, etc. Because our cohort does not represent a “normal” population, we cannot rule out the possibility that heterozygosity for this deletion is enriched in our patient population for a different reason, such as predisposing to phenotypic abnormalities. However, this is unlikely given the fact that heterozygous individuals are asymptomatic and that homozygous individuals have only hearing loss and, in males, infertility, as the only clinical manifestations. We also cannot rule out the possibility of a point mutation in *STRC* on the non-deleted allele; however such mutations are likely not as common as deletions [8], and array CGH testing is not typically performed on patients with hearing loss as a sole phenotypic finding. In addition, a recent publication reported a carrier frequency of 1.6%; however the cohort analyzed in this study was significantly smaller (n = 729) and combined multiple data sets [7]. Another recent publication reported a higher than expected incidence of homozygous deletion of the 15q15.3 region in a handful of individuals with hearing loss who were negative for *GJB2* mutation [8]. Taken together with our data set (n > 5000), we propose that DIS is a greatly under-diagnosed cause of hearing loss and should be included in the differential diagnosis for all individuals with hearing loss. Therefore, current molecular genetic panels testing for mutations that cause hearing loss should be expanded to include deletion/duplication analysis for this region of chromosome 15. Targeted mutation analysis, such as multiplex ligation-dependent probe amplification, could also be considered in the case of a targeted or tiered approach to genetic testing for hearing loss.

## Methods

Genomic DNA extraction for molecular cytogenetic studies was performed using the PureGene method (Qiagen Inc., Valencia, CA) on peripheral blood lymphocytes. DNA was resuspended in 1X Tris-EDTA (TE, pH 8.0) and the concentration was measured with a Nanodrop ND-100 Spectrophotometer (Thermo Fisher Scientific Inc., Wilmington, DE). To perform array CGH, 1 μg of patient genomic DNA was labeled with Cy5 and an equal amount of gender-matched control DNA was labeled with Cy3 using the Genomic DNA Labeling Kit (Agilent Technologies Inc., Santa Clara, CA) according to the manufacturer’s protocol. Each sample was purified using a Multiscreen PCR 96-well plate and eluted in TE. Equal volumes (19.5 μl) of both patient and control DNA were mixed along with Cot-1 DNA and 2X hybe buffer (Agilent Technologies Inc., Santa Clara, CA). The mixtures were denatured at 95°C for 3 minutes and then incubated at 37°C for 30 minutes. Each mixture was then applied to an Agilent 4 × 180 K oligonucleotide array CGH slide and incubated while rotating at 65°C for 24 hours. Slides were washed and scanned on a G2565CA Microarray Scanner System (Agilent Technologies Inc., Santa Clara, CA). Data was analyzed using DNA Analytics version 4.0 (Agilent Technologies Inc., Santa Clara, CA). This research was approved by the Mayo Clinic Institutional Review Board (IRB).

## Competing interests

The authors declare that they have no competing interests.

## Authors’ contributions

NLH analyzed the laboratory data, drafted, revised, and finalized the manuscript. UA analyzed the laboratory data and involved in manuscript preparation. PB collected and analyzed the laboratory data. NB, JW, and DB clinically examined the patient and collected the clinical data. All authors read and approved the final manuscript.
